# 2,2′-Dichlorodiethyl ether: MAK Value Documentation, addendum – Translation of the German version from 2023

**DOI:** 10.34865/mb11144e11_2ad

**Published:** 2026-06-30

**Authors:** Andrea Hartwig, MAK Commission

**Affiliations:** 1 Institute of Applied Biosciences, Department of Food Chemistry and Toxicology, Karlsruhe Institute of Technology (KIT) Adenauerring 20a, Building 50.41 76131 Karlsruhe Germany; 2 Permanent Senate Commission for the Investigation of Health Hazards of Chemical Compounds in the Work Area, Deutsche Forschungsgemeinschaft, Kennedyallee 40, 53175 Bonn, Germany. Further information: Permanent Senate Commission for the Investigation of Health Hazards of Chemical Compounds in the Work Area | DFG

**Keywords:** 2,2′-dichlorodiethyl ether, toxicity, irritation, MAK value, maximum workplace concentration, skin absorption, peak limitation

## Abstract

The German Senate Commission for the Investigation of Health Hazards of Chemical Compounds in the Work Area (MAK Commission) has re-evaluated the occupational exposure limit value (maximum concentration at the workplace, MAK value) for 2,2′-dichlorodiethyl ether [111-44-4] considering all toxicological end points. Relevant studies were identified from a literature search. In a study from 1933, 2,2′-dichlorodiethyl ether in concentrations of 100 ml/m^3^ and above showed irritating effects in humans. At 35 ml/m^3^ a clearly perceptible unpleasant odour was described and this concentration was irritating in guinea pigs. Reduced body weight gain was observed in rats in a chronic feeding study. This effect was more pronounced in female animals. In a combined repeated dose toxicity study screening also for reproduction/developmental toxicity in rats, the NOAEL for systemic effects was 15 mg/kg body weight and day, the highest dose tested. On this basis, the MAK value has been set at 0.5 ml/m^3^. As the critical effect of 2,2′-dichlorodiethyl ether is systemic, Peak Limitation Category II has been assigned with an excursion factor of 2. As substances with a higher irritation potency like monochloroacetic acid and 2-chloroethanol have similar or higher MAK values, the MAK value for 2,2′-dichlorodiethyl ether will also protect from irritation. Limited data show no genotoxic or carcinogenic potential for 2,2′-dichlorodiethyl ether. In a screening study, the NOAEL for perinatal toxicity in rats was 15 mg/kg body weight and day. As teratogenicity was not investigated, 2,2′-dichlorodiethyl ether has been assigned to Pregnancy Risk Group D. 2,2′-Dichlorodiethyl ether is not a skin sensitizer in a Local Lymph Node Assay in mice. According to skin absorption models, 2,2′-dichlorodiethyl ether is expected to be taken up via the skin in toxicologically relevant amounts. Therefore, 2,2′-dichlorodiethyl ether remains designated with “H”.

**Table:** 

**MAK value (2022)**	**0.5 ml/m^3^ ≙ 3.0 mg/m^3^**
**Peak limitation (2022)**	**Category II, excursion factor 2**
	
**Absorption through the skin (1976)**	**H**
**Sensitization**	**–**
**Carcinogenicity**	**–**
**Prenatal toxicity (2022)**	**Pregnancy Risk Group D**
**Germ cell mutagenicity**	**–**
	
**BAT value**	**–**
	
Synonyms	bis(2-chloroethyl) ether β,β′-dichloroethyl ether
Chemical name (IUPAC)	1-chloro-2-(2-chloroethoxy)ethane
CAS number	111-44-4
Structural formula	ClH_2_C–CH_2_–O–CH_2_–CH_2_Cl
Molecular formula	C_4_H_8_Cl_2_O
Molar mass	143.01 g/mol
Melting point	–51.9 °C (NCBI 2021)
Boiling point at 1013 hPa	172–178 °C (ECHA 2022)
Density at 20 °C	1.22 g/cm^3^ (NCBI 2021)
Vapour pressure at 25 °C	2.06 hPa (NCBI 2021)
log K_OW_	1.29 (NCBI 2021)
Solubility	10 200 mg/l water (NCBI 2021)
**1 ml/m^3^ (ppm) ≙ 5.934 mg/m^3^**	**1 mg/m^3^ ≙ 0.169 ml/m^3^ (ppm)**
	
Hydrolytic stability	stable at 30 °C for > 24 hours, pH 7 (Van Duuren et al. 1972)
Uses	solvent for fats, waxes and esters; in cleaning oils and petrol; in paints and varnishes; microbicide; corrosion inhibitor in the petroleum industry; wetting, cleaning and finishing agent for textiles (ATSDR 2017)

For 2,2′-dichlorodiethyl ether, documentation is available from 1977 (Henschler 1977, available in German only), and there are addenda available for the end points carcinogenicity (Henschler 1978, 1984, available in German only) and peak limitation (Greim 2002, available in German only).

All end points are re-evaluated in this addendum. The addendum is based in part on the publicly available registration data from the European Chemicals Agency ECHA (2022) and summaries of the toxicological data from ATSDR (2017).

## 1 Toxic Effects and Mode of Action

2,2′-Dichlorodiethyl ether is absorbed completely after ingestion and metabolized, to inter alia chloroethanol and chloroacetaldehyde.

2,2′-Dichlorodiethyl ether concentrations of 100 ml/m^3^ and above cause irritation in humans and have a nauseating odour. In guinea pigs, nasal irritation occurred after single exposures at and above 35 ml/m^3^, and lung congestion, oe­dema and haemorrhage, reduced respiratory rate and motor abnormalities were observed at and above 105 ml/m^3^. After exposure to 69 ml/m^3^ for 130 days, body weight gains were reduced in rats and guinea pigs. After oral administration for 78 weeks, a study of limited validity revealed reduced body weights in female and male CD rats at 25 and 50 mg/kg body weight and day and increased mortality in female animals at 50 mg/kg body weight and day.

Undiluted 2,2′-dichlorodiethyl ether is not irritating to rabbit skin and was not irritating to chicken eyes in an in vitro study.

2,2′-Dichlorodiethyl ether was found to be mutagenic in bacteria and mammalian cells in vitro, in the latter at highly cytotoxic concentrations. DNA adducts do not occur with 2,2′-dichlorodiethyl ether in rat liver. Negative results in a test for heritable translocations in Drosophila and a micronucleus test in mouse lymphoma cells in vitro did not provide any evidence of clastogenicity.

Carcinogenic potential cannot be deduced from the available studies with oral administration in rats or after dermal application and intraperitoneal and subcutaneous injection in mice. There is no evidence of sensitization in humans or animals.

## 2 Mechanism of Action

The adverse effects are regarded to be the result of the reactivity of the chlorine atoms of the substance and the supposed metabolite chloroacetaldehyde with cell components. It has been suggested that the toxicity occurs when inactivation of the substance by glutathione has been exhausted (BUA 1992).

Chloroacetaldehyde is formed also in the metabolism of vinyl chloride. However, it can be ruled out that 2,2′-dichlorodiethyl ether has, like vinyl chloride, a carcinogenic effect, as the responsible metabolite chloroethylene oxide is not formed in the metabolism of 2,2′-dichlorodiethyl ether. DNA adducts were not found in rats after the administration of either 2,2′-dichlorodiethyl ether or chloroethanol, which is likewise metabolized to chloroacetaldehyde (Gwinner et al. 1983; Henschler 1984). Although chloroacetaldehyde forms DNA adducts in vitro (Greim 1999), it can be assumed that chloroacetaldehyde is only short-lived in the metabolism of 2,2′-dichlorodiethyl ether and is rapidly detoxified in vivo.

The hepatomas observed in a long-term study from 1969, which can be evaluated only to a limited extent (see Section 5.7.2), are regarded as a species-specific effect in the mouse strains used, which are susceptible to this type of tumour (Laube et al. 2019; Maronpot 2009). Following subcutaneous injection, sarcomas formed on the skin of mice at a low incidence (Van Duuren et al. 1972); these tumours were presumably the result of irritant effects, as a potential for tumour initiation on the skin of mice following dermal application could not be derived for 2,2′-dichlorodiethyl ether (Van Duuren et al. 1972).

## 3 Toxicokinetics and Metabolism

### 3.1 Absorption, distribution and elimination

There are no data in humans available. Data for the metabolism of the substance have to date been reported only in summarized form. All the available data are described below.

#### 3.1.1 Inhalation

The blood:air partition coefficient according to the formula of Buist et al. (2012) is 15 800.

Three male Wistar rats weighing about 200 g were exposed in a closed glass chamber for 24 hours to ^14^C-2,2′-dichlorodiethyl ether (1.42 mCi/mmol), which was applied to a filter paper placed in the glass chamber (no other details) and evaporated. According to the authors, more than 95% of the applied amount was taken up by the animals within 18 hours, which corresponded to 0.25 mCi and thus about 50 mg per animal. This was calculated by means of the decrease in the concentration of 2,2′-dichlorodiethyl ether in the air of the glass chamber, which was determined by gas chromatography. Based on the body weight data, the total dose was 250 mg/kg body weight. There are no other data available (Gwinner et al. 1983).

Five groups of 8 male Wistar rats weighing 200–250 g were exposed separately to 2,2′-dichlorodiethyl ether concentrations of 0, 10, 50, 100 or 500 ml/m^3^ in inhalation chambers for 8 hours (no other details). The urine collected within 24 hours was prepared and the amount of the main metabolite (thiodiglycolic acid) excreted was determined by gas chromatography. The 2,2′-dichlorodiethyl ether concentrations administered of 0, 10, 50, 100 and 500 ml/m^3^ yielded excreted amounts of about 0.05, 0.1, 0.2, 0.21 and 0.27 mg, respectively. Further data for distribution and excretion are not available (Norpoth et al. 1986).

#### 3.1.2 Oral administration

Two female rhesus monkeys weighing 8.9 and 9.75 kg were given a single ^14^C-2,2′-dichlorodiethyl ether dose of 10 mg/kg body weight with the diet. The radioactivity in the collected urine was determined after 6, 12, 24, 36, 48, 72 and 96 hours, and that in the faeces after 24, 48, 36, 72 and 96 hours. In the 96-hour period after administration, only 1.1% and 1.63% of the administered radioactivity was excreted in the faeces. Within 48 hours, 51% and 61% of the radioactivity was determined in the urine (ECHA 2022).

Seven male Wistar rats weighing 180–240 g were given 40 mg 2,2′-dichlorodiethyl ether per kg body weight in corn oil by gavage. Thiodiglycolic acid was identified as a metabolite in the urine (see Section 3.2.2). In the expired air, 1.6% was excreted as the initial substance within 8 hours after administration. No exhaled 2,2′-dichlorodiethyl ether was detected in the period from 8 to 48 hours after the start of the study. The main route of excretion was the urine, as 65% of the administered substance was detected in the 48-hour urine, primarily in the form of thiodiglycolic acid (Lingg et al. 1979).

The results described above were further analysed in follow-up experiments with the same experimental design. This time, the starting material was labelled (1-^14^C-2,2′-dichlorodiethyl ether) and ^14^CO_2_ was determined in addition. Within 48 hours, 64.7% of the administered dose was excreted in the urine, 2.4% in the faeces and 11.5% as ^14^CO_2_. Radioactivity bound in organs and tissues totalled 2.3% of the administered dose. In the muscles, kidneys, blood and liver, 0.96%, 0.56%, 0.49% and 0.19% of the administered dose were detected, respectively. The total amount recovered was reported to be 80.9% of the administered radioactivity. A total of 78.6% of the administered dose was excreted within 48 hours and the half-life, based on the cumulative excretion of the substance in the urine and expired air, was found to be 12 hours (Lingg et al. 1982). As only 2.4% was found in the faeces, it can be assumed that 2,2′-dichlorodiethyl ether is absorbed almost completely after oral administration.

#### 3.1.3 Dermal application

No experimental data are available. Model calculations with the IH SkinPerm model according to Tibaldi et al. (2014) or according to Fiserova-Bergerova et al. (1990) result in a total absorbed amount of 50 mg or 417 mg, respectively, for the exposure of 2000 cm^2^ of skin to a saturated aqueous solution of the substance for 1 hour.

#### 3.1.4 Summary

2,2′-Dichlorodiethyl ether is rapidly absorbed and distributed after inhalation and oral administration in animal studies. Based on the results of acute dermal toxicity studies (see Section 5.1.3), dermal absorption can be assumed. In view of the low quantities excreted with the faeces, absorption can be assumed to be almost complete after oral administration. High bioaccumulation is not to be expected, as about 80% of the administered radioactivity is excreted 48 hours after oral administration to rats and the half-life for cumulative excretion in the urine and expired air is 12 hours (ATSDR 2017). However, it is not possible to determine the exact half-life based on the available data. Excretion of the substance occurs mainly in the form of metabolites in the urine and as exhaled carbon dioxide.

### 3.2 Metabolism

#### 3.2.1 Inhalation

Wistar rats excreted thiodiglycolic acid, but not 2-hydroxyethylmercapturic acid (as found in parallel experiments with vinyl chloride), as a metabolite in the urine (Norpoth et al. 1986).

#### 3.2.2 Oral administration

After an oral 2,2′-dichlorodiethyl ether dose of 40 mg/kg body weight in corn oil, thiodiglycolic acid was detected as the main metabolite and 2-chloroethyl-β-glucuronic acid as a further metabolite in the 48-hour urine of rats. Based on stoichiometric calculations, 77.6% or 38.8% of the initial substance was assumed to be converted during metabolism to thiodiglycolic acid with subsequent conjugation with glutathione. The calculation is based on the assumption that one molecule of 2,2′-dichlorodiethyl ether is metabolized to either 1 or 2 molecules of thiodiglycolic acid. The detection of thiodiglycolic acid and its glucuronide indicates that 2-chloroethanol is formed in the metabolism of 2,2′-dichlorodiethyl ether. In the exhaled air collected, 1.6% of the administered dose was determined within 8 hours as the initial substance (Lingg et al. 1979).

In another study with the same experimental design and using radioactively labelled substance, the excreted metabolites were determined by gas chromatography. Analysis of the 48-hour urine of 7 test animals revealed the main metabolite to be thiodiglycolic acid with a proportion of about 75% of the radioactivity (48% of the administered radioactivity). Other metabolites were 2-chloroethoxyacetic acid (about 3% of the administered radioactivity) and *N*-acetyl-*S*-[2-(2-chloroethoxy)ethyl]-*L*-cysteine (about 4.5% of the administered radioactivity). Metabolism therefore proceeds via hydroxylation and subsequent reactions via the presumed intermediates 2-chloroethanol and chloroacetaldehyde to form chloroacetic acid, which is conjugated with glutathione and further converted to thiodiglycolic acid. Furthermore, oxidative dehalogenation of one of the terminal chlorine atoms to form 2-chloroethoxyacetic acid and substitution with glutathione and subsequent degradation to *N*-acetyl-*S*-[2-(2-chloroethoxy)ethyl]-*L*-cysteine can be assumed (Lingg et al. 1982). The formation of the glucuronide of 2-chloroethanol as observed by Lingg et al. (1979) was not detected in this experiment.

**Fig. 1:**
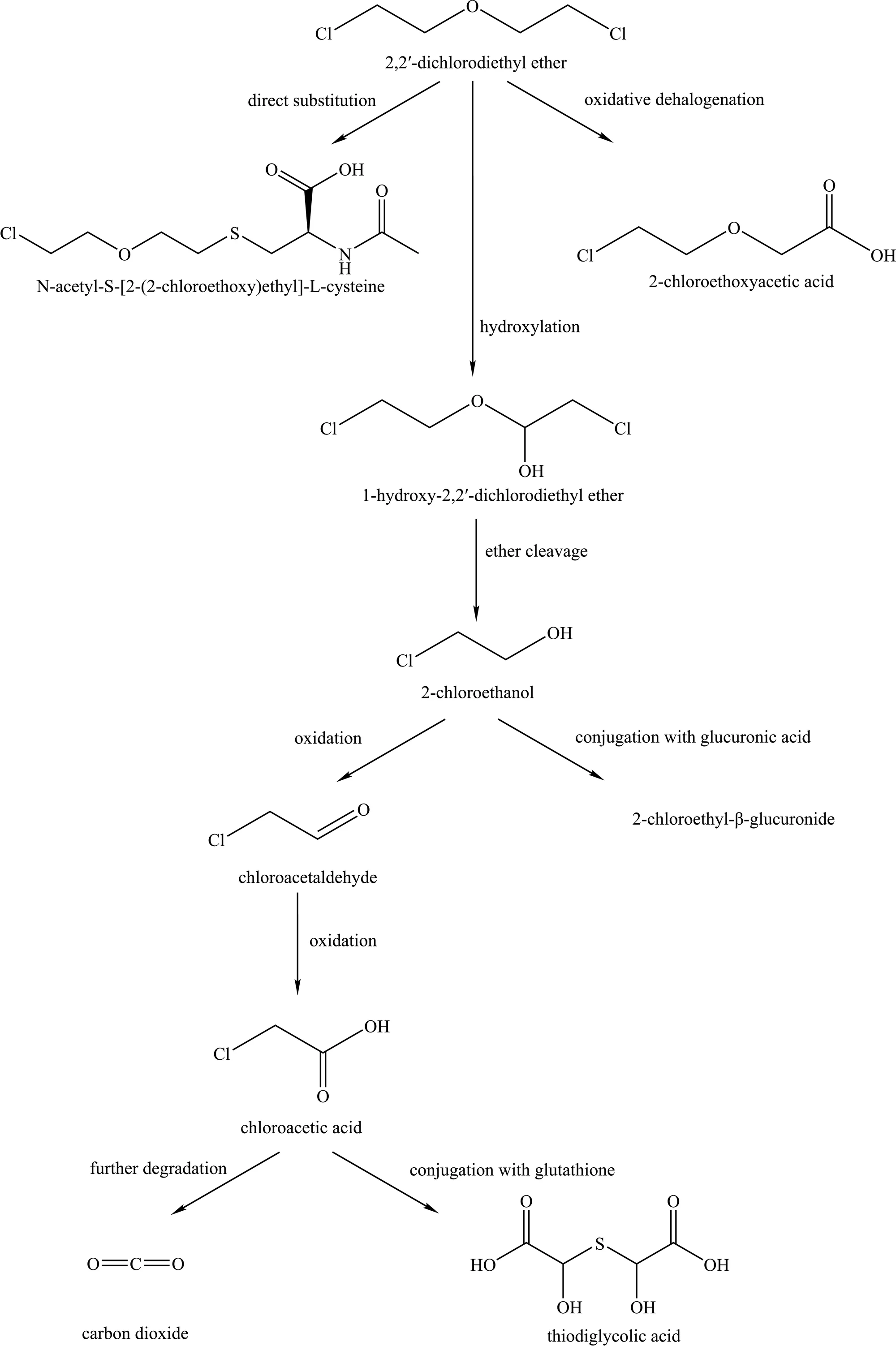
Postulated metabolism of 2,2′-dichlorodiethyl ether in rats, adapted according to ATSDR (2017)

A diagram of the metabolism after oral administration according to ATSDR (2017) is shown in Figure 1.

#### 3.2.3 Intraperitoneal injection

Male Wistar rats with an average weight of 200 g were given an intraperitoneal injection of 100 mg 2,2′-dichlorodiethyl ether/kg body weight (purity: 99%). Urine was collected for 24 hours after the injection and analysed. The 2 metabolites detected were thiodiglycolic acid and *S*-(carboxymethyl)-*L*-cysteine, which is a precursor of thiodiglycolic acid (Müller et al. 1979).

Five male Wistar rats weighing 200–250 g were given intraperitoneal injections of 2,2′-dichlorodiethyl ether (purity: 99%) dissolved in sunflower oil. The doses were 40 and 160 µmol 2,2′-dichlorodiethyl ether/kg body weight (5.7 and 22.8 mg 2,2′-dichlorodiethyl ether/kg body weight, respectively). In the 24-hour urine, 2.9 and 6.5 mg thiodiglycolic acid and 0.51 and 0.72 mg 2-hydroxyethyl mercapturic acid were recovered, respectively (Norpoth et al. 1986).

#### 3.2.4 Summary

2,2′-Dichlorodiethyl ether is almost completely metabolized in rats. Only small amounts (< 2% of the administered dose) are eliminated unchanged in the expired air after oral administration. The main metabolite in the urine is thiodiglycolic acid (about 50% of the administered radioactivity after 48 hours). Metabolism proceeds by hydroxylation via the intermediates 2-chloroethanol and chloroacetaldehyde (which, themselves, have not yet been detected) to chloroacetic acid, which is conjugated with glutathione and further converted to thiodiglycolic acid. Furthermore, oxidative dehalogenation of one of the terminal chlorine atoms to form 2-chloroethoxyacetic acid and direct substitution with glutathione and subsequent degradation to *N*-acetyl-*S*-[2-(2-chloroethoxy)ethyl]-*L*-cysteine is possible. Formation of the glucuronide 2-chloroethyl-β-glucuronic acid, presumably starting from the intermediate 2-chloroethanol, has also been shown (Lingg et al. 1979). Another metabolite produced is CO_2_. 2-Hydroxyethylmercapturic acid was detected in rats in small amounts after intraperitoneal injection, but not after inhalation (Norpoth et al. 1986).

## 4 Effects in Humans

No epidemiological studies are available for 2,2′-dichlorodiethyl ether. There are insufficiently documented data for the acute effects of 2,2′-dichlorodiethyl ether in humans. 2,2′-Dichlorodiethyl ether concentrations of 550 and 1000 ml/m^3^ led to intolerable irritation of the eyes and nose together with lacrimation and nausea after brief inhalation exposure in volunteers (no other details). At concentrations of 260 and 100 ml/m^3^, this irritation was less pronounced and the odour was described as slightly nauseating. After exposure to a 2,2′-dichlorodiethyl ether concentration of 35 ml/m^3^, the study participants still reported a slightly perceptible, unpleasant odour but almost no irritant effects (no other details; Schrenk et al. 1933). In this study, the concentration in the exposure room was determined, but there is no information on the purity of the substance and possible impurities in the breathing air cannot be ruled out. Due to the inadequate description of the effects and the study procedure, these data can be used for evaluation only to a very limited extent.

A concentration of 15 mg/m^3^ (2.55 ml/m^3^) for a 2 minute-exposure is given as the threshold for the irritation of the mucous membranes and eyes of humans in an overview of limit values in the former Soviet Union (Izmerov et al. 1982). Further data for this specification are not available and therefore this value cannot be used for the evaluation of 2,2′-dichlorodiethyl ether.

There are no sufficiently documented reports of injuries caused by 2,2′-dichlorodiethyl ether during industrial handling. One death has been documented, probably due to the inhalation of a vapour mixture containing 2,2′-dichlorodiethyl ether among other substances. However, there are no other data available (Henschler 1977).

The data for the odour threshold for 2,2′-dichlorodiethyl ether vary greatly. Amoore and Hautala (1983) give a value of 0.049 ml/m^3^, while Ruth (1986) reports values between 15 and 400 ml/m^3^. Odour perception in the range of the MAK value cannot be ruled out. The odour quality is specified in the PubChem database as “pungent, nauseating, sweet, solvent like” (NCBI 2021). The available information provides no evidence of relevant chemosensory effects in the range of the MAK value.

There are no data available for the reproductive toxicity, carcinogenicity and allergenicity of 2,2′-dichlorodiethyl ether.

## 5 Animal Experiments and in vitro Studies

### 5.1 Acute toxicity

The following data have become available since the publication of the previous documentation (Henschler 1977).

#### 5.1.1 Inhalation

In inhalation studies with rats, mice, guinea pigs and rabbits (no other details), death occurred within 1 to 2 hours (ATSDR 2017). In rats, the LC_50_ after exposure for 4 hours was 250 ml 2,2′-dichlorodiethyl ether/m^3^. Four of 6 guinea pigs died after inhalation exposure to 105 ml/m^3^ for 13 hours. Respiratory distress, and oedema and haemorrhage in the lungs occurred within 1 to 5 hours. Nasal irritation was observed after exposure to 35 ml/m^3^, which occurred after only 10 minutes (Schrenk et al. 1933). In this study, the concentration was determined in the exposure room, but there is no information on the purity of the substance and possible impurities in the breathing air cannot be excluded. For rats and mice (no other details), LC_50_ values of 330 and 650 mg 2,2′-dichlorodiethyl ether/m^3^, respectively, were reported after inhalation exposure for 4 hours (NCBI 2021).

#### 5.1.2 Oral administration

Oral LD_50_ values reported for rats were 75 and 144 mg 2,2′-dichlorodiethyl ether/kg body weight, for mice 211 mg/kg body weight and for mice, rats and rabbits values were given in the range of 105–136 mg/kg body weight (ATSDR 2017). The purity of the test substance was not specified.

#### 5.1.3 Dermal application

The LD_50_ values reported after dermal exposure of rabbits and guinea pigs were 870 and 370–390 mg 2,2′-dichlorodiethyl ether/kg body weight, respectively (ATSDR 2017). No information is available on the details of the exposure and the purity of the test substance. The lowest value of 9 mg/kg body weight for rabbits is given as a worst–case estimate, as it is not clear from the available data whether the LD_50_ value of 90 mg/kg body weight given by the study authors refers to the 10% solution in propylene glycol or to the pure substance (ECHA 2022).

### 5.2 Subacute, subchronic and chronic toxicity

#### 5.2.1 Inhalation

In an earlier study, groups of 15 male and 15 female rats and groups of 8 male and 8 female guinea pigs were exposed to 2,2′-dichlorodiethyl ether concentrations of 0 or 69 ml/m^3^ (no other details). Exposure took place for 7 hours daily, on 5 days per week, for 130 days (equivalent to 92 exposure days). The lungs, heart, liver, kidneys and testes were examined histopathologically and the organ weights (no other details) were determined. The organs and the urine and blood parameters examined did not reveal any substance-related effects and behaviour was also unaffected. Body weight gains were decreased in both species (no other details; ATSDR 2017). No irritant effects were reported.

#### 5.2.2 Oral administration

To determine the maximum tolerated dose for a combined repeated dose and reproductive toxicity study according to OECD Test Guideline 422 (see Section 5.5.2), groups of 3 female and 3 male Sprague Dawley rats were given gavage doses of 2,2′-dichlorodiethyl ether of 0, 1, 5, 20 or 100 mg/kg body weight and day for 14 days. The purity of the test substance was 99.5%. At 20 and 100 mg/kg body weight and day, a low respiratory rate and low spontaneous locomotion were observed in the animals. In addition, weight loss, staggering gait, abnormal gait, coma, a low stool amount, low body temperature, lid closure and deep breathing were observed at 100 mg/kg body weight and day. The animals remained mainly in a recumbent position and exhibited staining of the lower abdomen and around the anus. All animals died at this dose. The maximum dose for the main test was set at 15 mg 2,2′-dichlorodiethyl ether/kg body weight and day (CERI Hita 2007).

In the study according to OECD Test Guideline 422, 12 male and 12 female Sprague Dawley rats per dose group were treated by gavage with 2,2′-dichlorodiethyl ether (purity 99.5%) dissolved in olive oil. The 2,2′-dichlorodiethyl ether doses were 0, 0.6, 3 or 15 mg/kg body weight and day. Dosing of males began 14 days before the mating period. The total duration of treatment of the male and female rats, including the mating, gestation and lactation periods (up to day 5 of lactation), was 42 and 42–45 days, respectively. In the parental generation, statistically significant decreases in erythrocyte volumes and haemoglobin levels were observed at the end of the treatment period in males given 3 mg/kg body weight. In female animals of the 0.6 mg/kg group, a statistically significant increase in lymphocyte counts was observed. After the recovery period, a statistically significant increase in the number of red blood cells, a statistically significant decrease in the number of reticulocytes and neutrophils, and a statistically significant increase in the nitrogen content of the blood were observed in the females of the 15 mg/kg group. In the male animals of the high dose group, increased blood chloride levels and statistically significant increases in the absolute and relative weights of the adrenal glands were observed at the end of the recovery phase. In female animals of this dose group, a statistically significant increase in the absolute weights of the thymus gland was determined. In individual animals of the control group and some exposed animals, effects on the gastric mucosa and the forestomach were observed. All these findings were considered toxicologically irrelevant, coincidental and not substance-related due to a lack of dose dependence and a lack of further associated effects in histopathology. A NOAEL of 15 mg/kg body weight and day was given (CERI Hita 2007).

In a 78-week study, groups of 26 female and 26 male CD-1 rats were given gavage doses of 2,2′-dichlorodiethyl ether of 0, 25 or 50 mg/kg body weight in vehicle solution (sodium chloride, carboxymethylcellulose sodium, polysorbate and benzyl alcohol in water) twice a week. A commercially available test substance was used for this purpose. According to the authors, contamination of the test solution cannot be ruled out. The animals were followed up for 26 weeks before they were killed and examined. Reduced body weights (no other details) were observed in female rats at and above 25 mg/kg body weight and in male rats at 50 mg/kg body weight. In addition, mortality was increased in female animals after 52 weeks at 50 mg/kg body weight. In the control group without treatment and in the vehicle controls, 98% and 96% of females and 96% and 82% of males survived, respectively (see Section 5.7.2; Weisburger et al. 1981). A NOAEL could not be determined, the LOAEL (lowest observed adverse effect level) was 25 mg 2,2′-dichlorodiethyl ether/kg body weight. The study can be used for evaluation only to a limited extent because the authors noted that the dose used was possibly too low and additionally detailed data from the pathological examination were not reported.

#### 5.2.3 Dermal application

There are no data available.

### 5.3 Local effects on skin and mucous membranes

#### 5.3.1 Skin

In an unpublished study from 1980, the 4-hour occlusive application of 0.5 ml undiluted 2,2′-dichlorodiethyl ether (purity not specified) to the clipped skin of 6 New Zealand White rabbits (3 males and 3 females) did not cause any irritant effects on the skin up to 48 hours after the end of application. At 4, 24 and 48 hours, the irritation scores for erythema and oedema were 0 on a scale with a maximum of 4 according to Draize. Because of this earlier study, which was conducted before the introduction of GLP or OECD Test Guideline 404, the REACH registration dossier does not include a classification according to GHS for skin irritation (ECHA 2022).

After a single “non-occlusive” application (no other details) of 10 mg 2,2′-dichlorodiethyl ether (purity not specified), irritation of the skin of rabbits was observed (ATSDR 2017; BUA 1992). As no further information is available, this study cannot be used for the evaluation.

In a local lymph node assay (LLNA), 3 applications of 50% 2,2′-dichlorodiethyl ether (purity not specified) in acet­one/olive oil (4:1 v/v) to the ears of 4 female CBA/Ca mice did not cause any irritant effects at the application site (ECHA 2022).

#### 5.3.2 Eyes

In an earlier study it was reported that immediately after the start of exposure (no other details) to concentrations of 2,2′-dichlorodiethyl ether of 550 or 1000 ml/m^3^ in guinea pigs (no other details), lacrimation and squinting of the eyes occurred. Exposure to 260 ml 2,2′-dichlorodiethyl ether/m^3^ resulted in squinting within 1 minute and lacrimation after 3 minutes. Lacrimation was not observed after exposure to 105 ml 2,2′-dichlorodiethyl ether/m^3^ even after 810 minutes of exposure, but after 20 minutes the animals reacted by squinting their eyes. None of the above effects were observed after exposure for 810 minutes to 35 ml/m^3^ (Schrenk et al. 1933). In this study, the concentration in the exposure room was determined, but there is no information on the purity of the substance and possible impurities in the breathing air cannot be ruled out.

Moderate irritation of the eyes of rabbits was observed after the instillation of 25 mg of 2,2′-dichlorodiethyl ether in a 0.02 ml solution (purity not specified). The observation period was 18 to 24 hours. The eyes were not rinsed. The grade of severity was given as 4 on a scale with a maximum of 10. It is not reported whether the effects were reversible (no other details, Carpenter and Smyth 1946). The authors stated that many of the 180 substances they tested were contaminated industrial chemicals. This may explain the contradictions between the results of this study and those of the in vitro study with isolated chicken eyes described below, in which no eye irritation was observed.

In an in vitro study, 2,2′-dichlorodiethyl ether (purity > 99%) was tested in isolated chicken eyes in accordance with OECD Test Guideline 438. Either 30 µl saline solution (negative control), benzalkonium chloride solution (positive control) or 2,2′-dichlorodiethyl ether (100%) was dropped into each of 3 chicken eyes. After 10 seconds, all eyes were washed with 20 ml saline and examined 30, 75, 120, 180 and 240 minutes later. The parameters were the swelling rate of the cornea, the mean maximum opacity score and the mean fluorescein retention score. The mean values for corneal swelling (up to 240 minutes) were 1.6, for corneal opacity and fluorescein discoloration 0.17. The respective ICE classification scales for corneal swelling, the degree of opacity and fluorescein retention ranged from 0 to more than 32, 0 to 3 and 0 to 4, respectively. All 3 results of the tests with 2,2′-dichlorodiethyl ether were assigned to ICE class I on the basis of the evaluation scale and thus did not lead to classification according to the UN GHS classification system, which is made only at values of 2 or 3 and higher. The positive and negative controls indicated that the test system functioned correctly (ECHA 2022). Further in vitro tests have not been performed.

#### 5.3.3 Summary

In an earlier skin irritation study, occlusive application of undiluted 2,2′-dichlorodiethyl ether (purity not specified) for 4 hours did not produce any irritant effects on shaved rabbit skin. In a study from the 1940s, a moderate irritant effect on the rabbit eye was observed, but it is unclear whether this effect was due to impurities. In a current in vitro study with chicken eyes conducted according to OECD Test Guideline 438, an irritant effect on the eyes could not be derived for undiluted 2,2′-dichlorodiethyl ether (purity > 99%).

### 5.4 Allergenic effects

#### 5.4.1 Sensitizing effects on the skin

In an LLNA according to OECD Test Guideline 429 with 4 female CBA/Ca mice per concentration group, stimulation indices of 1.3, 1.0 and 1.1 were achieved with 10%, 25% and 50% test preparations in acetone/olive oil (4:1 v/v). The test results were therefore negative (ECHA 2022).

#### 5.4.2 Sensitizing effects on the airways

There are no data available for sensitizing effects on the airways.

### 5.5 Reproductive and developmental toxicity

#### 5.5.1 Fertility

In a combined repeated dose toxicity study with a screening test for reproductive/developmental toxicity according to OECD Test Guideline 422, male and female Sprague Dawley rats were treated with gavage doses of 2,2′-dichlorodiethyl ether (purity 99.5%) dissolved in 5 ml olive oil. A pre-test to determine the maximum dose for the main test is described in Section 5.1. The daily doses in the main study were 0 (vehicle control), 0.6, 3 or 15 mg 2,2′-dichlorodiethyl ether/kg body weight and day. Twelve female and 12 male animals were used per dose. Dosing of the males began 14 days before the mating period. The total duration of treatment of the male and female rats, including the mating, gestation and lactation periods (up to day 5 of lactation), was 42 and 42–45 days, respectively. No substance–related effects on the reproductive organs and oestrus cycle were observed in the parental generation. In addition, no effects were observed on the gestation or implantation index or on the number of implantation sites. A statistically significant decrease in the delivery index (number of offspring born/number of implantation sites × 100) was reported at 0.6 and 15 mg/kg body weight and day, but not at 3 mg/kg body weight and day. A statistically significant lower birth index (number of live offspring on postnatal day 0/number of implantation sites × 100) was observed at 0.6, 3 and 15 mg/kg body weight and day (84.4%, 83.0% and 81.3%, respectively). This value was 92.8% in the control group. It was not possible to conclude whether the effect was due to toxicity to the germ cells or embryotoxicity (CERI Hita 2007). The results of the study are shown in Table 1. The NOAEL for parental toxicity and fertility was 15 mg/kg body weight and day, the highest dose tested.

In an earlier study, groups of 15 male and 15 female rats and 8 male and 8 female guinea pigs (no other details) were exposed to 2,2′-dichlorodiethyl ether concentrations of 0 or 69 ml/m^3^ (purity not specified) for 7 hours daily, on 5 days a week, for 130 days. No effects on the testes were observed (ATSDR 2017). The original study is not available and is not suitable for evaluation due to limitations in the study design and documentation.

#### 5.5.2 Developmental toxicity

In the combined repeated dose toxicity study and screening test for reproductive toxicity according to OECD Test Guideline 422 (for effects on the parent animals see Sections 5.2.2 and 5.5.1), increased post-implantation losses were observed even at the lowest dose tested of 0.6 mg/kg body weight and day. The reduced indices (delivery index, birth index) have already been mentioned (see Section 5.5.1 and Table 1).

The ratio of live offspring to the total number of animals born at 3 mg/kg body weight and day was 88.8%; compared with the value in the control group (96%) this represented a statistically significant decrease. As this effect was not observed at the dose of 15 mg/kg body weight and day, it was classified as coincidental. Survival on postnatal day 4 (98.1%) was higher in the dose group given 0.6 mg/kg body weight and day compared with that in the control group (89.9%), whereas the survival rate in the group given 3 mg/kg body weight and day was only 80.1%. No significant effect was reported in the high dose group. Since a dose–response relationship could not be derived, this effect was likewise categorized as coincidental (CERI Hita 2007). The results of the study are shown in Table 1. The NOAEL for maternal toxicity is 15 mg/kg body weight and day, the highest dose tested.

In studies carried out in accordance with OECD Test Guideline 422, only an external examination is performed on the offspring, but not a skeletal or visceral examination. Teratogenic effects are therefore not fully investigated.

Both parameters “delivery index” and “birth index” were analysed in relation to the number of implantation sites, which is very unusual. According to OECD Test Guideline 422, the recommended parameters for recording the offspring are the number of dams with live offspring, the number of live offspring per dam on the day of birth (average) and litter size.

The increase in post-implantation losses was dose-dependent and statistically significant in the chi-square test. In the 0.6 mg/kg body weight dose group, the animal that died before birth was incorrectly included in the post-implantation losses. However, the parameter was not analysed on a litter basis, which is why the post-implantation losses were subsequently calculated on a litter basis by the Commission. The post-implantation losses were first calculated for each litter from the data of the original study as the difference between the number of implantation sites and the number of live offspring in relation to the number of implantation sites multiplied by 100. From this, mean values and standard deviations were calculated for all dose groups, resulting in post-implantation losses per litter of 7.11% ± 10.67%, 8.77% ± 9.85%, 16.35% ± 17.86% and 18.19% ± 19.68% for the control group and the individual doses in ascending order. The subsequent statistical analysis with Dunnett’s test did not lead to statistically significant differences in the pairwise comparison with the controls (p < 0.05). Therefore, the Commission does not assume developmental toxicity here.

Historical control values of the testing laboratory for post-implantation losses are not included in the original study report. For Crl:CD(SD) rats, a value of 4.9% (range: 2.0% to 9.9%) can be found for post-implantation losses per litter from 168 studies (WIL Research Laboratories) for the years 1998 to 2010 (Stump et al. 2012).

**Tab. 1:** Combined toxicity study with screening test for reproductive/developmental toxicity of 2,2′-dichlorodiethyl ether with Sprague Dawley rats (12 female rats per dose group and control) (CERI Hita 2007)

	**Dose [mg/kg body weight and day]**			
	**0**	**0.6**	**3**	**15**
**Parameter**				
Number of mated, copulating animals	12	12	12	12
Number of pregnant animals	12	12	12	12
Copulation index (%)^k)^	100	100	100	100
Fertility index (%)^l)^	100	100	100	100
Conception index (%)^m)^	100	100	100	100
Mating period before copulation (days) (MV ± SD)	2.7 ± 1.0	2.2 ± 1.0	2.0 ± 1.0	2.0 ± 1.0
Gestation period (days) (MV ± SD)	21.9 ± 0.5	21.9 ± 0.3	22.1 ± 0.5	22.2 ± 0.4
Number of dams (PND 0)	12	11	12	12
Number of dams (PND 4)	11	11	9	11
Number of corpora lutea	15.3	15.9	15.7	15.7
Number of implantation sites	15.1	15.5	15.2	15.2
Gestation index (%)^a)^	100 (12/12)	81.7 (11/12)	100 (12/12)	100 (12/12)
Implantation index (%)^b)^	98.9 (181/183)	97.4 (186/191)	96.8 (182/188)	96.8 (182/188)
Delivery index (%)^c)^	96.7 (175/181)	87.1 (162/186)*	93.4 (170/182)	88.5 (161/182)*
PND 0:				
Number of offspring born (MV ± SD)	14.6 ± 1.6	14.7 ± 1.7	14.2 ± 1.6	13.4 ± 3.1
Number of live offspring (MV ± SD)	14.0 ± 2.2	14.3 ± 2.2	12.6 ± 2.9	12.3 ± 3.1
Number of live male offspring (MV ± SD)	6.9 ± 2.1	7.3 ± 2.1	6.9 ± 2.9	6.3 ± 2.6
Number of live female offspring (MV ± SD)	7.1 ± 2.7	7.0 ± 2.1	5.7 ± 2.1	6.1 ± 2.4
Birth index^d)^ (%)	92.8 (168/181)	84.4 (157/186)*	83.0 (151/182)**	81.3 (148/182)**
Live birth index^e)^ (%)	96.0 (168/175)	96.9 (157/162)	88.8 (151/170)*	91.9 (148/161)
Sex ratio^f)^	83/168	80/157	83/151	75/148
Sex ratio per dam (MV ± SD)	0.5 ± 0.15	0.51 ± 0.12	0.54 ± 0.15	.51 ± 0.15
Pre-implantation losses (%)^g)^	1.1 (2/183)	2.6 (5/191)	3.2 (6/188)	3.2 (6/188)
Post-implantation losses (%)^h)^	7.2 (13/181)	15.6 (29/186)*	17.0 (31/182)**	18.7 (34/182)**
Weight of male offspring [g] (MV ± SD)	6.4 ± 0.5	6.6 ± 0.5	6.6 ± 0.6	6.5 ± 0.7
Weight of female offspring [g] (MV ± SD)	6.2 ± 0.5	6.3 ± 0.5	6.0 ± 0.6	6.3 ± 0.7
PND 4:				
Number of live offspring (MV ± SD)	13.7 ± 1.8	14.0 ± 2.1	13.4 ± 2.1	11.8 ± 3.0
Number of live male offspring (MV ± SD)	6.5 ± 1.9	7.1 ± 1.9	7.8 ± 2.5	5.9 ± 2.2
Number of live female offspring (MV ± SD)	6.7 ± 3.1	7.0 ± 2.1	4.3 ± 3.0	5.4 ± 2.8
Viability index^i)^ (%)	89.9 (151/168)	98.1 (154/157)**	80.1 (121/151)*	87.8 (130/148)
Sex ratio^j)^	71/151	78/154	70/121	65/130
Sex ratio per dam (MV ± SD)	0.48 ± 0.13	0.51 ± 0.12	0.57 ± 0.13	0.50 ± 0.16
Weight of male offspring [g] (MV ± SD)	9.6 ± 1.7	10.3 ± 1.3	9.8 ± 2.1	11.0 ± 1.2
Weight of female offspring [g] (MV ± SD)	9.3 ± 1.6	9.9 ± 1.2	9.1 ± 2.1	10.5 ± 1.6

MV: mean value; SD: standard deviation

* p < 0.01;

** p < 0.05

a) Number of pregnant animals with live offspring/number of pregnant animals × 100

b) Number of implantation sites/number of corpora lutea × 100

c) Number of offspring born/number of implantation sites × 100

d) Number of live offspring on PND 0/number of implantation sites × 100

e) Number of live offspring on PND 0/number of offspring born × 100

f) Number of live male offspring on PND 0/number of live offspring on PND 0

g) Number of corpora lutea – number of implantation sites/number of corpora lutea × 100

h) Number of implantation sites – number of live offspring on PND 0/number of implantation sites × 100

i) Number of live offspring on PND 4/number of live offspring on PND 0 × 100

j) Number of male offspring on PND 4/number of live offspring on PND 4

k) Number of copulating pairs/number of mated pairs

l) Number of males that led to fertilization/number of males from copulating pairs

m) Number of pregnant female animals/number of female animals from copulating pairs

#### 5.5.3 Summary

In a combined repeated dose study with a screening test for reproductive/developmental toxicity according to OECD Test Guideline 422 in male and female Sprague Dawley rats, a statistically significant increase in post-implantation losses on a foetal basis, but not on a litter basis, was found even at the low dose of 0.6 mg/kg body weight and day (subsequent statistical analysis). The NOAEL for maternal toxicity was 15 mg/kg body weight and day, the highest dose tested.

### 5.6 Genotoxicity

#### 5.6.1 In vitro

##### 5.6.1.1 Bacteria and yeasts

There was no evidence of RNA strand breaks in studies with the bacteriophage R17 (Shooter 1975). Without the add­ition of a metabolic activation system, no SOS response was induced in Escherichia coli MT119 and no recombinations occurred in Escherichia coli MT119 and MT126. Treatment of the Escherichia coli strains MT103 and MT126 did not lead to increased mutation frequencies (Quinto and Radman 1987). There is no information on the concentrations used.

2,2′-Dichlorodiethyl ether was investigated in plate tests with bacteria (Salmonella typhimurium strains TA98, TA100, TA1535, TA1537; Escherichia coli WP2 uvr^–^) and yeast (Saccharomyces cerevisiae D3) both in and not in a desiccator. In the pre-incubation test, 2,2′-dichlorodiethyl ether had a weak mutagenic effect on Salmonella typhimurium TA100 without metabolic activation (no other details). On the other hand, a pronounced mutagenic effect was observed after exposure in the desiccator; that is, exposure of the bacteria plated on agar to the substance in the gaseous phase (Simmon et al. 1977). Further results from these studies are not reported and information on how the experiments were carried out, the evaluation of the results and the controls are only incompletely described. The study is therefore only of limited use for the evaluation.

In another study, mutagenic effects of the substance on Salmonella typhimurium TA100 and TA1535 and Escherichia coli 343/113 in liquid culture were not observed either with or without the addition of metabolic activation (Henschler 1977).

In parallel studies by 2 laboratories, the mutagenicity of 2,2′-dichlorodiethyl ether in Salmonella typhimurium was examined in a pre-incubation test. A mutagenic effect was observed in strain TA100 without metabolic activation after tests by Case Western Reserve University but not after tests by SRI International Laboratories. According to the authors, the inconsistencies are due to different test conditions and procedures between the 2 laboratories. These include differences in the concentrations used, the age of the cell cultures and the composition of the metabolic activation as well as the incubation times and test temperatures (Mortelmans et al. 1986).

In another Salmonella mutagenicity test, 2,2′-dichlorodiethyl ether led to a concentration-dependent increase in revertants in the presence of a metabolic activation system in strain TA100 even at the lowest concentration, which was already cytotoxic. At the highest concentration tested of 40 µg/plate, there was a doubling of the mutants. Additional irradiation with UV light had no effect on this result. A positive control was not included in the tests; the solvent was methanol (Norpoth et al. 1986).

In a mutagenicity test with pre-incubation in the Salmonella typhimurium strains TA98, TA100, TA1535, TA1537 and TA1538 from 1989, no mutagenic effects were found either with or without metabolic activation. Concentrations were tested up to the range of cytotoxicity (ECHA 2022). The substance was labelled as DCEE (dichloroethyl ether). It is therefore not clear whether this refers to 2,2′-dichlorodiethyl ether.

A mutagenic effect of 2,2′-dichlorodiethyl ether was reported in a study with Escherichia coli, Bacillus subtilis and Salmonella typhimurium, which is available only as an abstract (ECHA 2022). As the study was published only in summarized form, it cannot be used for the evaluation.

In a mutagenicity test according to the current OECD Test Guideline 471, the effects of 0, 78.1, 156.2, 312.5, 625, 1250, 2500 and 5000 µg 2,2′-dichlorodiethyl ether were examined using plate incorporation and pre-incubation. The tests were performed with the Salmonella strains TA98, TA100, TA1535, TA1537 and with Escherichia coli WP2 uvrA. Only vehicle controls and no untreated negative controls were analysed. In the plate incorporation test with Escherichia coli WP2 uvrA, however, the mutation frequency was increased only up to 2.12-fold and 2.78-fold. No cytotoxic effects were observed in any of the strains in the plate incorporation test. The pre-incubation test revealed a mutagenic effect on TA1535 with metabolic activation at 1250 and 2500 µg 2,2′-dichlorodiethyl ether per plate. The number of revertants was increased 3.18-fold and 4.91-fold (the positivity criterion for this strain is 3). A concentration–dependent increase in revertants was observed also with Escherichia coli WP2 uvrA, but the increase was less than 2-fold (positivity criterion for this strain). Cytotoxicity was observed with and without the addition of a metabolic activation system at 5000 µg 2,2′-dichlorodiethyl ether per plate for all strains tested and at and above 2500 µg without metabolic activation for strain TA1537 (ECHA 2022). The presentation of results in the registration dossier does not contain precise information on the number of revertants.

##### 5.6.1.2 Mammalian cells

In a micronucleus test in L5178Y mouse lymphoma cells according to OECD Test Guideline 487, the cells were exposed for 3 hours with the addition of a metabolic activation system and then examined after 24 hours or exposed without a metabolic activation system for 3 or 24 hours and analysed directly. Exposure was carried out without the addition of a metabolic activation system with 2,2′-dichlorodiethyl ether concentrations of 0, 250, 1000 or 2000 µg/ml. In tests with the addition of a metabolic activation system, 2,2′-dichlorodiethyl ether concentrations of 0 or 2.5 to 10 µg/ml were used. Although micronuclei were induced at the tested concentrations, the increase was neither concentration–dependent nor statistically significant compared with the control value. The test result was therefore regarded as negative. In tests without the addition of a metabolic activation system, cytotoxicity was observed at and above 2,2′-dichlorodiethyl ether concentrations of 500 µg/ml and in tests with metabolic activation at and above 10 µg/ml (ECHA 2022). The presentation of results in the registration dossier does not contain any information on the micronucleus frequency. In addition, the expiry date of the substance used is stated as 1 June 2016, but the test period lasted until 11 July 2016.

In a TK^+/–^ test in L5178Y mouse lymphoma cells carried out according to OECD Test Guideline 490, the mutagenic effects were investigated after an exposure period of 3 hours with the addition of a metabolic activation system or an exposure period of 3 or 24 hours without metabolic activation. The 2,2′-dichlorodiethyl ether concentrations used for preparations with metabolic activation and incubation for 3 hours were 0, 1.25, 2.5, 5, 10, 20, 40, 60, 80 and 120 µg/ml. The 2,2′-dichlorodiethyl ether concentrations used for preparations without metabolic activation (incubation for 3 or 24 hours) were 0, 46.88, 93.75, 187.5, 375, 750 and 1500 µg/ml. Pronounced cytotoxicity (relative total growth: about 16%–19%) after metabolic activation was observed at 2,2′-dichlorodiethyl ether concentrations of 20 µg/ml and above. Without metabolic activation, mutagenicity or cytotoxicity were not observed. After exposure with metabolic activation for 3 hours, a concentration-dependent mutagenic effect was observed, which, however, was statistically significant and biologically relevant (difference to the negative control < “global evaluation factor”) only at the highest evaluated concentration, at which excessive cytotoxicity occurred (ECHA 2022). The expiry date of the substance used is given as 1 June 2016, but the test period extended until 2 February 2017. No distinction was made between small and large colonies.

#### 5.6.2 In vivo

##### 5.6.2.1 Drosophila

Evidence of mutagenicity was not observed in Drosophila melanogaster (Henschler 1977).

In an SLRL test (sex-linked recessive lethal mutations) in Drosophila melanogaster, negative results were obtained when male Canton S flies were exposed to 2,2′-dichlorodiethyl ether in the diet for 3 days before mating with female Basc flies, whereas positive results were obtained after injection. A subsequent heritable reciprocal translocation test carried out in Drosophila after the injection of 13 000 mg 2,2′-dichlorodiethyl ether/kg, performed due to the positive result after injection in the above-mentioned test, yielded a negative result (Foureman et al. 1994). In a test for somatic mutations and recombinations (SMART; eye mosaic test) with inhalation exposure of the larvae, the number of spots per 100 evaluated eyes at the highest concentration tested, which was lethal for 99% of the larvae, was only slightly more than double that in the concurrent controls (Ballering et al. 1996).

##### 5.6.2.2 Mammalian cells

Studies of the interaction of the substance with DNA were carried out with rats. Three male Wistar rats were exposed whole-body to ^14^C-2,2′-dichlorodiethyl ether applied to filter paper in an inhalation chamber over a period of 24 hours. The total amount absorbed was 0.25 mCi/animal (equivalent to 50 mg ^14^C-2,2′dichlorodiethyl ether). The covalently bound radioactivity was then determined in the lungs, liver, kidneys, spleen, small intestines and muscle tissue. Binding to proteins was highest in the liver, followed by the kidneys and small intestines. After purification and hydrolysis of liver DNA and RNA, no evidence was found for the formation of the alkylation products 7-*N*(2-oxoethyl)guanine, 1,*N*^6^-ethenoadenine or 3,*N*^4^-ethenocytosine, which are typical of vinyl chloride and are held responsible for its carcinogenic effects, and the corresponding chromatograms did not yield evidence of other adducts (Gwinner et al. 1983).

A test for hereditary translocations in adult mice (no other details) yielded a negative result. The male parental animals received the substance for 8 weeks by gavage at 3 different dose levels (no other details; Jorgenson et al. 1978). As the study was published only as an abstract, it cannot be used for the evaluation.

#### 5.6.3 Summary

2,2′-Dichlorodiethyl ether induces mutations in bacteria in non-cytotoxic concentrations. In mouse lymphoma cells, no micronuclei are induced, whereas TK^+/–^ mutations are induced but only at concentrations that already have a very strong cytotoxic effect.

In Drosophila, the result of a SLRL test was positive after injection of a high dose, whereas the result was negative after oral administration, as was a test for hereditary translocations.

Overall, the database for genotoxicity is insufficient. The findings in vitro cannot be conclusively evaluated as corres­ponding results from studies of mutagenic effects in vivo are not available. However, no DNA adducts were detected in the liver of rats after exposure to 2,2′-dichlorodiethyl ether. A negative test result for hereditary translocations in Drosophila and a negative micronucleus test in mouse lymphoma cells in vitro do not provide any indication of a clastogenic effect.

### 5.7 Carcinogenicity

Previous studies for this end point were described in the documentation and the addenda (Henschler 1977, 1978, 1984). Carcinogenic effects could not be derived from these data. In addition, although chloroacetaldehyde is formed metabolically from both vinyl chloride and 2,2′-dichlorodiethyl ether, a carcinogenic effect analogous to that of vinyl chloride was not observed (Henschler 1984). All carcinogenicity studies are summarized and evaluated below.

#### 5.7.1 Short-term studies

The formation of preneoplastic ATPase-deficient areas in the liver of rats was investigated after exposure to vinyl chloride or 2,2′-dichlorodiethyl ether. For this purpose, 25 or 50 mg 2,2′-dichlorodiethyl ether/kg body weight dissolved in a commercially available coffee cream was administered orally to 6 male and 5 female Wistar rats using a pipette. Treatment took place daily for 5 days. The control animals received only the vehicle. After 10 weeks, the animals were killed and sections of the liver were examined. The formation of preneoplastic ATPase-deficient areas was not observed in the rat liver. In contrast, such areas occurred in the rat liver together with typical DNA adducts (see Section 5.6.2) in animals exposed to vinyl chloride by inhalation (Gwinner et al. 1983). Since no DNA adducts and foci were detected in this study after exposure to 2,2′-dichlorodiethyl ether, the suspected carcinogenicity, analogous to that after exposure to vinyl chloride, has been disproven (Henschler 1984).

In an initiation–promotion study, the initiating potential of 2,2′-dichlorodiethyl ether was investigated on the skin of mice. For this purpose, 1 mg of the substance, dissolved in 0.1 ml benzene, was applied once to the skin of 20 female, 6-week-old ICR/Ha mice by micropipette. Assuming a weight of 20 g per animal, a dose of 50 mg/kg body weight can be calculated. The promotion treatment began 2 weeks after the initiation treatment. Each animal was treated 3 times a week at the application site with 2.5 µg phorbol myristate acetate (PMA) dissolved in 0.1 ml acetone. A further group of 20 animals received no initiation treatment, but only PMA. Another group of 20 animals received only acetone. An additional control group, consisting of 60 mice, remained untreated. All surviving animals were examined 590 days after the start of treatment with PMA or acetone. Three of the 20 mice treated with 2,2′-dichlorodiethyl ether and PMA developed a papilloma on the skin, and in the PMA-only group, 2 mice developed a papilloma. In the group treated with acetone and the completely untreated animals, no papillomas formed (Van Duuren et al. 1972). 2,2′-Dichlorodiethyl ether was therefore not tumour–initiating in mouse skin.

#### 5.7.2 Long-term studies

##### 5.7.2.1 Inhalation

There are no data available.

##### 5.7.2.2 Oral administration

Seven-day-old mice of 2 strains (C57BL/6 × C3H/Anf and C57BL/6 × AKRF_1_) were given gavage doses of 2,2′-dichlorodiethyl ether of 100 mg/kg body weight dissolved in water daily for 3 weeks. A commercially available test substance was used for this purpose; information on the purity is not available. This dose corresponded to the maximum tolerated dose. The animals were then given a 2,2′-dichlorodiethyl ether dose of 300 mg/kg diet until the age of 80 weeks, which corresponds to about 45 mg/kg body weight and day (conversion factor 0.15 for the mouse (chronic exposure) according to EFSA (2012)). For each strain and sex, 18 animals per dose group and several control groups were used. Five different positive controls with administration of amitrole, aramite, dihydrosafrole, isosafrole and safrole were included. The doses were administered first by gavage for 3 weeks and then with the diet for 80 weeks. All surviving and prematurely deceased animals were examined gross pathologically and their main organs (no other details) histologically. All tissues with abnormalities were likewise included in the examination. The results are shown in Table 2. Compared with the findings in the untreated control group, a statistically significant increase in the incidence of hepatomas was observed in the animals exposed to 2,2′-dichlorodiethyl ether of strain C57BL/6 × C3H/Anf and in the male animals of strain C57BL/6 × AKRF_1_. Increased mortality was observed in males of the C57BL/6 × C3H/Anf strain (Innes et al. 1969). Due to several factors, the study can be used for evaluation only to a limited extent. Only one dose (maximum tolerated dose) was used and the number of animals was low. The tumour findings were also insufficiently described. A differentiated, quantitative distinction between adenomas and carcinomas is not given. The control animals were introduced into the experiments at different times and the time to necropsy was in the range of 78–89 weeks and was therefore different to that of the exposed animals, which were sacrificed at 80 weeks of age. The increased mortality of males of the C57BL/6 × C3H/Anf strain is not explained and further limits the evaluation of the findings in this group. The hepatomas are regarded as a species-specific effect in these mouse strains, which are susceptible to these tumours (Laube et al. 2019; Maronpot 2009).

**Tab. 2:** Carcinogenicity of 2,2′-dichlorodiethyl ether after oral administration

Author:	Innes et al. 1969				
Substance:	2,2′-dichlorodiethyl ether (purity: commercial product, no other details)				
Species:	mouse C57BL75 × C3H/Anf and C57BL75 × AKRF_1_, 18 animals per dose and sex				
Administration route:	oral, for 3 weeks, gavage; oral, for 76 weeks, in the diet				
Dose:	100 mg/kg body weight and day, 21 days by gavage (7-day-old animals); at the age of 4 weeks about 300 mg 2,2′-dichlorodiethyl ether/kg diet (about 45 mg/kg body weight and day)^a)^				
Duration:	79 weeks				
Toxicity:	mortality: ↑ in male C57BL/6 × C3H/Anf animals				
		**Dose [mg/kg body weight and day]**			
		**0**	**0**	**100**	**100**
**Strain**		**C57BL75 × C3H/Anf**	**C57BL75 × AKRF_1_**	**C57BL75 × C3H/Anf**	**C57BL75 × AKRF_1_**
surviving animals^b)^	♂	73/90 (81%)	89/90 (99%)	11/18 (61%)	15/18 (83%)
	♀	83/90 (92%)	75/90 (83%)	18/18 (100%)	17/18 (94%)
**Tumours:**					
**Liver:**					
hepatomas	♂	8/79 (10%)	5/90 (6%)	14/16 (88%)*	9/17 (53%)*
	♀	0/87 (0%)	1/82 (1%)	4/18 (22%)*	0/18 (0%)
**Lungs:**					
tumours (no other details)	♂	5/79 (6%)	10/90 (11%)	0/16 (0%)	2/17 (12%)
	♀	3/87 (3%)	3/82 (4%)	0/18 (0%)	0/18 (0%)
**Lymphatic system:**					
lymphomas	♂	5/79 (6%)	1/90 (1%)	2/16 (13%)*	0/17 (0%)
	♀	4/87 (5%)	4/82 (5%)	0/18 (0%)	1/18 (6%)
**Total number of animals with “tumours”**	♂	22	16	16	10
	♀	8	7	4	1

* p < 0.01

a) calculated using a conversion factor of 0.15 for the mouse, chronic exposure (EFSA 2012)

b) controls after 78–89 weeks, exposed groups at 80 weeks of age

Another long-term study was conducted with CD1 rats. For this purpose, groups of 26 female and 26 male animals were given gavage doses of 2,2′-dichlorodiethyl ether of 0, 25 or 50 mg/kg body weight dissolved in vehicle solution (sodium chloride, carboxymethylcellulose sodium, polysorbate and benzyl alcohol). A commercially available test substance was used for this purpose. According to the authors, contamination of the test solution cannot be ruled out. Treatment took place twice a week for a period of 78 weeks, followed by an observation period of 26 weeks after which the animals were examined. The control groups consisted of 184 male and female rats without treatment and 58 male and female rats that were given only the vehicle. Information on the age of the animals at the start of treatment is not available. Animals that died in the meantime were also examined. The examination included gross pathology and the detailed histopathological examination of the following organs and tissues: cerebrum, cerebellum, pituitary gland, spinal cord, lungs, heart, mediastinum, thymus, thyroid gland, parathyroid gland, liver, pancreas, spleen, adrenal gland, kidneys, bladder, ovaries, uterus, testes, genitals, oesophagus, stomach, digestive tract and all tissues with abnormalities (no other details). Mortality (65% surviving animals) was increased in the females at 50 mg/kg body weight and day after 52 weeks compared with the incidence in the control group. In the control group without treatment and in the vehicle controls, 98% and 96% of the females and 96% and 82% of the males survived. The body weights (no other details) of the female rats were lower than those of the control animals at and above 25 mg/kg body weight as were the body weights of the male rats at 50 mg/kg body weight. Compared with the findings in the control animals, there was no statistically significant increase in the incidence of neoplasms in any treated animal group. However, the authors pointed out that the dose for the male animals might have been too low to demonstrate a carcinogenic effect (Weisburger et al. 1981). The study has numerous shortcomings that allow an evaluation only to a limited extent: in addition to the dose level, that may have been too low, detailed data from the pathological examination and other information are not available.

##### 5.7.2.3 Subcutaneous injection

Once a week, 30 female ICR/Ha mice (6 weeks old) were given subcutaneous injections of 1 mg 2,2′-dichlorodiethyl ether dissolved in 0.05 ml paraffin oil. The substance was purified for the experiments and its identity was checked using nuclear magnetic resonance spectroscopy. Although information on the weight of the animals is not available, the dose can be calculated as 50 mg/kg body weight once a week assuming a weight of 20 g per animal. A further 30 mice received subcutaneous injections of pure paraffin oil. All injections were given once a week for the lifetime of the animals (mean lifetime 656 days). As a further control, 60 mice remained untreated. All animals were examined after death. This included dissections of the injection site of all animals and the additional examination of organs and tissues of animals that exhibited gross abnormalities. Sarcomas were found at the injection site in 2 of 30 mice treated with 2,2′-dichlorodiethyl ether, but no neoplasms were found in other tissues and organs. No sarcomas occurred in the control groups (Van Duuren et al. 1972). The potency of the substance to form sarcomas on the skin is described as low (ECHA 2022).

In another long-term study, groups of 50 female and 50 male Sprague-Dawley rats (weighing 200–250 g) were given subcutaneous injections into the nape of the neck of 4.36 or 11.3 µmol 2,2′-dichlorodiethyl ether (3.1 mg or 9.4 mg 2,2′-dichlorodiethyl ether/kg body weight at 200 g) dissolved in 0.25 ml dimethyl sulfoxide once a week for a period of 2 years. The purity of the commercially available test substance was given as > 98%. Control groups of 35 male and 50 female animals received injections of the solvent only or no injections. Of the animals that died prematurely and those that survived after 2 years, only the organs and tissues of animals with gross abnormalities were examined histologically. No significantly increased incidences of benign and malignant tumours were found after administration of 2,2′-dichlorodiethyl ether compared with the findings in the control groups (Norpoth et al. 1986).

##### 5.7.2.4 Intraperitoneal injection

Twenty male A/St mice (6–8 weeks old) were given injections with 2,2′-dichlorodiethyl ether of 0, 8 or 20 mg/kg body weight dissolved in the vehicle tricaprylin 3 times a week for 8 weeks. The purity of the commercially available test substance was given as 95%–99.9%. The doses corresponded to 20% and 50% of the maximum tolerated dose according to a pre-test. The total dose was 192 mg and 480 mg 2,2′-dichlorodiethyl ether/kg body weight, respectively. A control group of 50 animals received only the vehicle. In addition, 20 mice were given 4 injections of the maximum tolerated dose of 40 mg 2,2′-dichlorodiethyl ether/kg body weight. The animals were killed 24 weeks after the first injection, their lungs were removed and the surfaces were examined by microscope for adenomas. Some adenomas were then also examined histologically (no other details). In the groups exposed to 8 or 20 mg 2,2′-dichlorodiethyl ether/kg body weight, 18 and 20 animals survived, respectively, and in the group exposed to 40 mg/kg body weight, 15 animals survived. In the control group, 46 animals survived. The incidence of lung adenomas, calculated as the number of tumours per mouse, was lower in the exposed animals than that in the examined control group (Theiss et al. 1977).

#### 5.7.3 Summary

In a study which can be evaluated only to a limited extent, undefined hepatomas were observed in mice of 2 strains after oral administration. However, a study with oral administration in rats could not confirm this finding. The formation of preneoplastic ATPase-deficient areas was not observed in the rat liver. As the formation of hepatomas was limited to 2 mouse strains that are particularly susceptible to this type of tumour (Laube et al. 2019; Maronpot 2009), a carcinogenic potential of 2,2′-dichlorodiethyl ether for humans cannot be derived from this finding. After subcutaneous injection, a low potency for sarcoma formation was observed in mouse skin, but not in rat skin; this tumour is presumably due to the irritant effect of the substance. Intraperitoneal injection did not lead to the formation of lung tumours in mice. All the studies described have shortcomings as regards the methodology, dosage and reporting of the findings. In the light of all data available to date, 2,2′-dichlorodiethyl ether is not regarded as carcinogenic.

### 5.8 Other effects

There are no studies available for immunological effects after inhalation or oral exposure. In an in vitro study, the mitogenesis of T and B cell lymphocytes of the spleen of BALB/c and C3H/He mice was investigated. Lipopolysaccharides and concanavalin A were used as mitogens. Inhibition of the mitogenesis of T cell lymphocytes was not detected but dose-dependent inhibition of the mitogenesis of B cell lymphocytes was observed at 3 × 10^-5^ mol/l (Sakazaki et al. 2001).

## 6 Manifesto (MAK value/classification)

Critical effects are general systemic toxicity in the form of reduced body weight gains in rats after chronic exposure and sensory irritation in volunteers.

**MAK value. **In an earlier study of limited validity, concentrations of 100 ml/m^3^ and above led to irritant effects in humans. At 35 ml/m^3^, a slightly perceptible, unpleasant odour and almost no irritant effects were reported (no other details; Schrenk et al. 1933). The information on skin and eye irritation in animals is contradictory, but the valid study data do not suggest corrosive or strong irritant effects. However, there are no valid studies with repeated inhalation exposure available which would allow irritation of the respiratory tract to be evaluated.

General systemic toxicity in the form of reduced body weights after chronic exposure of rats was observed at 25 mg/kg body weight and above (Weisburger et al. 1981). In a study with repeated daily oral administration according to OECD Test Guideline 422, a NOAEL of 15 mg/kg body weight and day was obtained in rats (CERI Hita 2007).

The following toxicokinetic data are taken into consideration for the extrapolation of this NOAEL to a concentration in workplace air: the daily exposure of the animals in comparison with the 5 days per week exposure at the workplace (7:5), the corresponding species-specific correction value for the rat (1:4), the measured oral absorption (100%), the body weight (70 kg) and respiratory volume (10 m^3^) of the person and the assumed 100% absorption by inhalation. In addition, extrapolation from subacute to chronic exposure (1:6) and extrapolation from experimental studies with animals to humans (1:2) are taken into consideration. The concentration calculated from this is 0.52 ml/m^3^. Using the preferred value approach, a MAK value of 0.5 ml/m^3^ (3.0 mg/m^3^) is obtained.

The 78-week study of Weisburger et al. (1981) in rats with a LOAEL (reduced body weights) of 25 mg/kg body weight and day is of limited validity (see Section 5.7.2.2), but yields the same MAK value: the same conversion steps are used, but the twice-weekly exposure (2:5) and extrapolation to a NAEL (no adverse effect level) (1:3) are taken into account. Extrapolation for time is not necessary.

This MAK value is below or in the range of the MAK values of more irritating substances such as 2-chloroethanol (2 ml/m^3^; Hartwig and MAK Commission^2022^) or monochloroacetic acid (0.5 ml/m^3^; Hartwig and MAK Commission^2020^). The MAK value therefore provides protection also against irritant effects.

**Peak limitation. **Due to the critical systemic effects, 2,2′-dichlorodiethyl ether is assigned to Peak Limitation Category II. An exact half-life has not been determined, therefore the default excursion factor of 2 is applied. The then permissible short-term concentration of 1 ml/m^3^ corresponds to the short-term concentration of monochloroacetic acid and is below the MAK value for 2-chloroethanol (see above). Therefore, the short-term concentration does not lead to irritant effects.

**Prenatal toxicity. **In a combined repeated dose study with a screening test for reproductive/developmental toxicity according to OECD Test Guideline 422 in male and female Sprague Dawley rats, a statistically significant increase in post-implantation losses on a foetal basis, but not on a litter basis, occurred even at the lowest dose tested of 0.6 mg/kg body weight and day (subsequent statistical analysis). This is therefore not to be considered a toxic effect on development. The NOAEL for maternal toxicity is 15 mg/kg body weight and day, the highest dose tested. Since a detailed investigation of teratogenicity is not performed in studies carried out according to Test Guideline 422 and other valid data on developmental toxicity are not available, 2,2′-dichlorodiethyl ether is assigned to Pregnancy Risk Group D.

**Carcinogenicity. **In 2 mouse strains, oral administration up to the maximum tolerated dose of 2,2′-dichlorodiethyl ether caused the formation of hepatomas. However, oral administration to rats did not confirm this finding and the formation of preneoplastic ATPase-deficient areas was not observed in the rat liver. Since hepatoma formation was limited to 2 mouse strains that are particularly susceptible to this type of tumour (Laube et al. 2019; Maronpot 2009) and the study has methodological limitations, a carcinogenic potential cannot be inferred on the basis of this study. After subcutaneous injection, a low potency for sarcoma formation was observed in mouse skin, but not in rat skin; this can be attributed to the irritant effect of the substance. Intraperitoneal injection did not lead to the formation of lung tumours in mice. All these studies described have shortcomings as regards the methodology, dosage and reporting of the findings. Overall, 2,2′-dichlorodiethyl ether has no carcinogenic potential and is not categorized as a carcinogen.

**Germ cell mutagenicity. **There are insufficient data available for genotoxicity. Studies of effects on the germ cells are not available. There is no corresponding evidence for in vivo mutagenicity that would allow a final evaluation of the positive findings in vitro. DNA adducts were not detected in the liver of rats after exposure to 2,2′-dichlorodiethyl ether. A negative test result for hereditary translocations in Drosophila and a negative micronucleus test result in mouse lymphoma cells in vitro provide no evidence of a clastogenic effect. Based on these data, 2,2′-dichlorodiethyl ether is not classified in one of the categories for germ cell mutagens.

**Absorption through the skin. **Data for the absorption of 2,2′-dichlorodiethyl ether through the skin are not available. Since acute toxicity was observed after dermal application, it can be assumed that the substance is absorbed through the skin. Based on model calculations, the dermal absorption of 50 mg (IH SkinPerm; Tibaldi et al. 2014) or 417 mg (Fiserova-Bergerova et al. 1990) is to be expected for exposure of both hands and forearms for 1 hour. After inhalation exposure for 8 hours at the level of the MAK value of 3 mg/m^3^, 30 mg is absorbed, assuming a respiratory volume of 10 m^3^ and complete pulmonary absorption. Accordingly, the possible contribution of dermal uptake to systemic toxicity is not negligible and the designation of 2,2′-dichlorodiethyl ether with an “H” (for substances which can be absorbed through the skin in toxicologically relevant amounts) has been retained.

**Sensitization. **There are no clinical findings and no positive results from animal experiments on the skin sensitizing potential of 2,2′-dichlorodiethyl ether. 2,2′-Dichlorodiethyl ether is therefore not designated with “Sh” (for substances which cause sensitization of the skin). Data for sensitizing effects on the airways are not available. 2,2′-Dichlorodiethyl ether is therefore not designated with “Sa” (for substances which cause sensitization of the airways).
